# 1360. Remdesivir in COVID 19 patients with kidney disease: A study on the renal function and outcomes

**DOI:** 10.1093/ofid/ofad500.1197

**Published:** 2023-11-27

**Authors:** Marie Yvette Barez, C R Y S T A L F A Y E G Lagura, Leano-Togonon Jennifer Ivy

**Affiliations:** Southern Philippines Medical Center, davao, Davao del Sur, Philippines; Southern Philippines medical center, DAVAO, Davao del Sur, Philippines; Southern Philippines medical center, DAVAO, Davao del Sur, Philippines

## Abstract

**Background:**

The COVID-19 pandemic has high mortality rates in patients with kidney disease. Remdesivir, an antiviral nucleotide prodrug, showed to shorten time to recovery in adults hospitalized with COVID 19. However, individuals with kidney disease were not included in clinical trials, thus when emergency use authorization was granted, it was recommended to avoid use in patients with eGFR < 30mL/min “unless benefit outweighs the risk”. In this study, we aim to determine the effect of Remdesivir on renal function and outcomes among COVID19 patients with kidney disease.

**Methods:**

This retrospective study included the following: >18 years old, RT-PCR confirmed COVID19 infection treated with Remdesivir and who were confirmed to have kidney disease.

**Results:**

There were 106 patients included; the mean age was 62.25±13.96 years old, more than half of the population were males (53%). The majority of the population had acute kidney injury (70.7%) while the rest had chronic kidney disease and 25% underwent renal replacement therapy. Most of them were classified as severe (66%) and critical (25%) COVID infection.

To determine effects on renal function laboratory parameters were determined at baseline and after completion of treatment. There was an overall significant improvement in eGFR (< 0.01), albuminuria (0.013) and in acid-base balance (0.003) and odds ratio showed that none of the demographic, clinical and laboratory profile significantly increased the chance of death in terms of overall clinical outcome.

In terms of overall clinical outcome, this study had a mortality rate of 4.9% as 5 out of 106 patients expired.

**Conclusion:**

Use of Remdesivir in patients with kidney disease was not associated with renal function deterioration. Contrary to concerns there was rather an overall significant improvement in eGFR, degree of albuminuria and acid-base balance after treatment regardless of their disease severity and its use in patients on hemodialysis have not shown any detrimental impact on mortality.

**Disclosures:**

**All Authors**: No reported disclosures

